# Topics of Public Interest

**Published:** 1916-10

**Authors:** 


### TOPICS OF PUBLIC INTEREST

**Statistics of Periodicals.** The Bureau of the Census issues the following statements, for the year 1914. Dailies, 2580 (2600 in 1909), aggregate circulation 28 million; Sunday papers 570 (520 in 1909), 16 millions; Tri-weeklies 84 (73 in 1909), 549 thousand, Semi-weeklies 583 (635 in 1909), 2½ millions; Weeklies 15,166 (15,097 in 1909), 50 millions; Monthlies 2,820 (2,491 in 1909), 79 millions; Quarterlies 500 (361 in 1909), 18 millions; All others 442 (364 in 1909), nine millions. The total number increased only from 22,141 in 1909 to 22,745 in 1909 but the aggregate circulation increased about 25%. The gross incomes of newspapers consisted of 99½ million dollars from subscriptions and sales and of 184 millions from advertising. For other periodicals, the gross incomes were 64 and 72 million dollars respectively. The reading public should appreciate that they pay less than half —and for most periodicals aside from the popular monthly magazines—only about a third of the cost of their reading matter. The strength and quality of periodicals in general, depends upon advertising support and this, in turn, depends upon the attention which readers pay to the advertisements. The only serious competitors against legitimate periodical advertising are the circular and the sign board.—both nuisances to the busy public.

**Bringing the Childless Home and the Homeless Child Together.** The State Charities Aid Assn. since 1898, has found homes for 2,366 children of whom 839 have been legally adopted. Supervision is continued to assure proper home surroundings and against "misfits" but 76% of the children remained in the homes first selected. This is a worthy philanthropy for which financial support is needed. The element of supervision is more important than might appear. Some years ago, we happened to observe a case in which the laws of a certain state practically established a system of peonage. Under certain restrictions, any family could secure a child servant, to be set free at a given age with a sum of $100. In another instance, two sisters were adopted by well-to-do families from an orphan asylum. One of the children wished to trace her real parents but the asylum either could not or would not give information and, as it developed later, refused information to the parents also, who had placed the children in the institution because of temporary financial reverses and who subsequently became quite prosperous. Largely through the determined efforts of the little girl, the family was ultimately reunited.

---

**Teutonic War Losses.** British compilations of Teutonic reports give the following totals for the first 25 months of war, up to Sept. 1, 1916, exclusive of naval and colonial forces: Killed 832,000; Prisoners 165,000; Missing 234,000; Wounded 2,144,000; Total 3,375,000. Newspaper statistics for the first ten months of the war placed the losses at more than double the totals for 25 months. Over two million dead German, Austrian and Turkish soldiers have been restored to life, and considerably over a million prisoners have escaped, if we are to reconcile the two sets of statistics. We have previously emphasized the fact that even this terrible war will not in any sense depopulate the countries of Europe, even if protracted.

---

**Telephone Merger.** Up to date the telephone situation seems to be about as follows: The Federal Co. has about 15,000 subscribers, about 9,000 of whom have Bell connection also. It is losing at the rate of about $400,000 a year, to compensate for which loss, about 12,000 additional subscribers would be required, not to mention the proportionately small but in the aggregate considerable expense due to the additional equipment and service, much of the equipment in instruments, wiring, etc., being already on hand. The Bell Co. has about 48,000 subscribers and with the 6,000 exclusive Federal subscribers, would have a total of 54,000. It charges about 50% more for its service than the Federal already but says it cannot survive the loss of an additional gain of 6,000 subscribers without further increase of rates to about 200% of the Federal Standard. It is proposed to put the matter of a merger in the form of a popular referendum. The average subscriber does not care whether the companies merge or whether one goes out of business. He does not care much whether connections are made by dial or by an operator. In fact, in our experience, the majority of persons would rather use two hours of time in telling someone else to do something than half an hour in doing it themselves, and would not object to the greater chance of error in the former method. The main problem is the matter of expense, aggregate and in relation to service, remembering that a la carte charges always tend to increase cost proportionate to the amount of a commodity or service received but, by inforcing economy, to reduce consumption so that the total cost is not proportionately increased. The medical profession, like many other kinds of business, must consider not only its expense for outgoing messages but the even greater economic loss of anything that discourages free use of telephones. The Postal Telegraph Co., obviously something more than an interested by-stander, cites the following rates: Buffalo under competitive service, $84,; under monopoly, Baltimore $174; San Francisco $171; Washington $156; Cincinnati $87; Denver $96; New Orleans $102. These cities correspond fairly well in population and area with Buffalo, especially if we consider the range of population growth of Buffalo corresponding to the existence of the dual system and the probable growth of Buffalo in the immediate future. They show two things: that a monopoly tends strongly toward an increase of rates but that such an increase is not necessary.

We ask our readers to exercise their influence and, if given an opportunity, their vote in exactly the same way that they would if we announced that, having absorbed another journal and being compelled to print another thousand copies of each issue, we should be compelled to increase our subscription rate to 150 or 200% of the present.

---

**Conservation of Paper.** The increased cost of paper is a serious economic problem to all branches of the printing and publishing business and, indirectly and directly, to the entire community. Under present conditions, use of paper means destruction of forests which is of still greater importance. We ask, therefore, whether you personally find the increased cost of paper appreciable or not, that you use all possible economy in this regard and, especially that you render used paper available for re-use. In Buffalo, it is not necessary to sell paper to accomplish the ultimate economic end, though we know of a very worthy minor charity that has been almost entirely supported by donations of old magazines. The city collections of waste paper, etc., largely support the work of disposing of rubbish.

---

**$125,000** is the estimate of the estate of the late Dr. John B. Murphy.

---

**Indian Centenarian.** A Cayuse Indian, Ayoushakatsagom, died Aug. 23 at Pendleton, Ore. aged 120.

---

**Sane Fourth of July.** No tetanus and no blinding were reported this year, the total casualties being 850 with 30 deaths.

---

**Priority of Women Physicians.** Dr. A. Jacobi (Med. Rev. of Revs., Sept., states that the first woman admitted to the New York State County Medical Society was Dr. Mary E. Greene, Feb. 13, 1871; 2d, Dr. Emily Blackwell, May 5; 3d, Dr. Celestia Loring, Sept. 4. A woman was previously admitted by a Kansas Society. (Note: An interesting historic review of this subject would be welcome).

---

**Dental Preparedness.** Dr. Wallace Seccombe of Toronto gave a lecture at the Hotel Statler, Buffalo, Sept. 12, on The Work of the Canadian Army Dental Corps. The meeting was under the auspices of the Buffalo Branch of the Preparedness League of American Dentists.

---

**David's Disease.** John Ridlon of Chicago, (Boston M. & S. Jour. Sept. 7) shows that Jean Pierre David, described caries of the vertebrae in a prize essay published the year before Percival Pott wrote a paper on palsy of the lower limbs. David's first case dates back to 1766. The further criticism is made that Pott's original paper did not recognize the relation of palsy of the lower limbs to vertebral disease, that at no time did he have a clear conception of the nature of what is commonly termed Pott's disease, that his statements as to clinical symptoms are inaccurate and his therapeutics entirely worthless. On the other hand, David clearly recognized that paralysis was due to lesions of the vertebrae, gave a good clinical description of what we call Pott's disease and recognized the pathologic condition as well as possible in the absence of bacteriologic knowledge.

---

**The University of Buffalo** inaugurated its 71st session by a joint meeting of all departments in Townsend Hall, Sept. 25. The newly established Arts Dept. will have about 325 students. Many of these are pre-medical students, the

Medical Dept. already requiring one year of college study and after next year two years, prior to matriculation.

**Infantile Paralysis.** Buffalo has had, up to Sept. 12 reports, eight cases and two deaths. Last summer there were 30 cases. So far as we can judge, the principal incidence in western N. Y. is in the southern tier of counties, where the proportionate number of cases is very high. In spite of optimistic rumors and claims, no progress has been made recently in regard to etiology and treatment.

**Prolonged Fast.** Dr. H. G. Huffman, an oculist and advocate of fasting as a nature cure of Youngstown, O. (not listed in Polk) died Sept. 7 in a hospital of that city. Two years ago, he fasted 47 days in camp, after having been told that he had only a short time to live and returned to a fair degree of health. Last year, he fasted 30 days. His present fast began June 30 in an open air camp. At the end of this period he attempted to eat but was unable to do so and died after 69 days altogether.

